# Cardiovascular disease (CVD) and associated risk factors among older adults in six low-and middle-income countries: results from SAGE Wave 1

**DOI:** 10.1186/s12889-018-5653-9

**Published:** 2018-06-20

**Authors:** Ye Ruan, Yanfei Guo, Yang Zheng, Zhezhou Huang, Shuangyuan Sun, Paul Kowal, Yan Shi, Fan Wu

**Affiliations:** 1 Shanghai Municipal Center for Disease Control and Prevention (Shanghai CDC), Shanghai, China; 2 World Health Organization, Geneva, Switzerland; 3 Research Institute for Health Sciences, Chiang Mai University, Chiang Mai, Thailand

**Keywords:** Cardiovascular diseases, Prevalence, Risk Factors, Low and middle income countries

## Abstract

**Background:**

Cardiovascular disease (CVD) is one of the leading causes of death worldwide. Our study aimed to investigate the prevalence of two conditions, angina and stroke, and relevant risk factors among older adults in six low- and middle- income countries(LMICs).

**Methods:**

The data was from World Health Organization (WHO) Study on global AGEing and adult Health (SAGE) Wave 1 in China, Ghana, India, Mexico, Russian Federation and South Africa. Presence of CVD was based on self-report of angina and stroke. Multivariate logistic regression was performed to examine the relationship between CVD and selected variables, including age, sex, urban/rural setting, household wealth, and risk factors such as smoking, alcohol drinking, fruit/vegetable intake, physical activity and BMI.

**Results:**

The age standardized prevalence of angina ranged from 9.5 % (South Africa) to 47.5 % (Russian Federation), and for stoke from 2.0% (India) to 6.1 % (Russia). Hypertension was associated with angina in China, India and Russian Federation after adjustment for age, sex, urban/rural setting, education and marital status (OR ranging from 1.3 [1.1-1.6] in India to 3.8 [2.9-5.0] in Russian Federation), furthermore it was a risk factor of stroke in five countries except Mexico. Low or moderate physical activity were also associated with angina in China, and were also strongly associated with stroke in all countries except Ghana and India. Obesity had a stronger association with angina in Russian Federation and China(ORs were 1.5[1.1-2.0] and 1.2 [1.0-1.5] respectively), and increased the risk of stroke in China. Smoking was associated with angina in India and South Africa(ORs were 1.6[1.0-2.4] and 2.1 [1.2-3.6] respectively ), and was also a risk factor of stroke in South Africa. We observed a stronger association between frequent heavy drinking and stroke in India. Household income was associated with reduced odds of angina in China, India and Russian Federation, however higher household income was a risk factor of angina in South Africa.

**Conclusion:**

While the specific mix of risk factors contribute to disease prevalence in different ways in these six countries – they should all be targeted in multi-sectoral efforts to reduce the high burden of CVD in today’s society.

## Background

Cardiovascular diseases (CVDs) are by far the leading cause of death in the world. An estimated 17.9 million people died from CVDs in 2015. Ischemic heart disease (IHD) and stroke were the top two leading causes of CVD health lost in each world region [1, 2]. By 2030 more than 22.2 million people will die annually from CVDs. Populations in low and middle income countries (LMICs) now contribute 75% of the CVD deaths, which leads to 7% reduction of gross domestic product(GDP) in these countries [3].

A larger proportion of the global burden of CVDs is now borne by LMICs than in high income countries, this is despite a comparatively lower burden from risk factors in low compared to high income countries [4–6]. Given the high prevalence of CVD among older adults in LMIC, the projected increases in this population will be a major challenge for the health care system. Twenty-three percent of the total global burden of disease(GBD) was attributed to disorders in people aged 60 years and older. The main contributors to disease burden were CVDs, accounting for 30.3% of the total burden in older people in 2010 [7]. Reliable and comparable analysis of risks to CVD is especially important for projecting future disease burden and for shaping disease prevention efforts.

A number of population-based studies from lower income countries have suggested that socio-demographic characteristics are associated with CVD, with increasing age, female sex and lower education consistently associated with higher prevalence of CVD. Some epidemiological evidence also suggests that CVD is associated with behavioral risk factors such as smoking, alcohol use, low physical activity levels, and insufficient vegetable and fruit intake, hypertension is also regarded as a very important risk factor for CVD. Independently or in combination, these risk factors present an opportunity for interventions to reduce future CVD burdens in ageing populations in LMIC.A number of large recent studies have compared CVD risks in higher and lower income countries, providing valuable and needed information about CVD and CVD risks [4–6]. However, the results of these studies may not be representative of the older adult population. For example, the Prospective Urban Rural Epidemiology (PURE) study sampling strategy and distributions provide less reliable estimates at older ages [8]. The World Health Organization Study on global AGEing and adult health (SAGE) is focused on older adults and use similar methodology across countries to improve comparability of important covariates and disease prevalence. Three of the countries overlap in PURE and SAGE (China, India and South Africa) where SAGE includes three additional middle income countries (Ghana, Mexico and the Russian Federation).

The aim of the present study was to investigate the prevalence of two main CVDs (angina, stroke) and behavioural risk factors and associated social-economic status (SES) factors among older adults using a unique data set with nationally representative samples in six low and middle income countries.

## Methods

### Sample and procedure

The data was from World Health Organization (WHO) Study on Global AGEing and adult health (SAGE) Wave 1, a longitudinal cohort study of ageing and older adults from 2007 to 2010 in six low- and middle-income countries (China, Ghana, India, Mexico, Russian Federation and South Africa) [9]. SAGE Wave 1 used face-to-face individual interviews to capture data. All six countries implemented multistage cluster sampling strategies which resulted in nationally representative cohorts of older adults (http://www.who.int/healthinfo/sage/SAGEWorkingPaper5_Wave1Sampling.pdf?ua=1). Response rates for SAGE countries were Mexico 51%, India 68%, Ghana 80%, Russian Federation 83%, South Africa 77% and China 93%. Examination of non-respondent data suggested non-significant differences on some covariates (data not shown). Data were obtained following application for access through http://apps.who.int/healthinfo/systems/surveydata/index.php/catalog.

SAGE has been approved by the World Health Organization's Ethical Review Board. Additionally, each partner organization obtained ethical clearance through their respective review bodies. All study participants signed informed consent.

### Measures

#### CVDs conditions

Two methods of assessing presence or absence of CVD were used. One was based on self-report of angina or stroke; and the second used an algorithm based on validated symptom-reporting methods to estimate and compare prevalence rates.

#### Sociodemographic variables

Socio-demographic variables contain age, sex, education, rural/urban residence, and income quintiles. Age was categorized into four groups: 50 to 59 years; 60 to 69 years; 70 to 79 years; and 80 years or older. Education level was classified into seven categories for analysis using an international classification scheme [10]. The income quintiles were generated using an asset-based approach- possession of assets and dwelling characteristics [11], with quintile 1(Q1) the quintile of the poorest households and quintile 5(Q5) the quintile of the richest.

#### Risk factors

##### Tobacco use

Tobacco use was assessed by self-report and included different forms (manufactured or hand-rolled cigarettes, cigars, cheroots or whether tobacco is smoked, chewed, sucked or inhaled), and frequency of smoking, snuffing or chewing in each day over the week before interview[12], classified into four groups: never smoker, not current smokers, smokers(not daily) and current daily smokers.

##### Alcohol consumption

Alcohol consumption was categorized into four groups: life time abstainer, non-heavy drinkers, infrequent heavy drinkers and frequent heavy drinkers according to the consumption number of standard drinks of beer, wine and or spirit, fermented cider, and other alcoholic drinks during the week before interview.

##### Physical activity

Physical activity was measured by the Global Physical Activity Questionnaire (GPAQ) and assessed intensity, duration, and frequency of physical activity in three domains: occupational, transport-related, and discretionary or leisure time. Based on a standard classification scheme, three categories were generated: low, moderate and high levels [13].

##### Fruit and vegetable consumption

Fruit and vegetable consumption was assessed according to the number of daily servings eaten – with each serving approximating 80 grams. Five or more servings were defined as sufficient daily intake (at least 400 grams per day), fewer than five servings were categorized as insufficient [14].

##### Hypertension

The definition of hypertension used was systolic blood pressure ≥140mmHg and/or diastolic blood pressure ≥ 90mmHg and/or self-reported treatment with antihypertensive medication during the two weeks before interview. Blood pressure measurements were conducted three times on the right arm of the seated respondent with an automated recording device (OMRON R6 Wrist Blood Pressure Monitor, HEM-6000-E, Omron Healthcare Europe), and calculated as an average of the latter two measurements.

##### Obesity

According to the classification criteria proposed by the WHO [15], body mass index (BMI) of <18.5 kg/m^2^, 25–29.9 kg/m^2^ and ≥30 kg/m^2^ are used to define underweight, overweight and obesity, respectively. Modified BMI cutoffs for China and India were used to perform an additional set of analyses that describes overweight (BMI 23.0-27.5) and obesity (BMI >27.5) in Asian populations [16].

### Statistical methods

Statistic analyse were conducted using STATA SE version 11 (Stata Corp, College Station, TX). The prevalence of angina and stroke were calculated by using normalized weights in each country. Weights were based on selection probability, non-response, and post-stratification adjustments. To improve comparability across countries, the prevalence rates were age-standardized using the WHO World Standard Population Distribution based on world average population 2000-2025 [17]. Multivariate logistic regression was performed to examine the relationship between CVD and selected variables, including the socio-demographics such as age, sex, urban/rural setting, education, household wealth, and health risk factors such as smoking, alcohol drinking, fruit/vegetable intake, physical activity, hypertension and obesity. *P* < 0.05 from two-sided statistical tests was considered statistically significant.

## Results

A total of 34,114 individuals were included in the final analyses. Table 1 shows the sample distribution and demographic, socioeconomic and lifestyle characteristics by countries. The proportions of women are higher than men in four countries, except Ghana and India. The majority of older Indian lived in rural locations, while compared to urban areas in the other countries. The 50-59 age groups had the largest proportions in all countries, but the SAGE sample population distributions match those of the United Nations and US Census Bureau’s International Data Base estimates [18]. The percentage of respondents with no formal education were higher in Ghana (54.0%) and India (51.2%). In contrast, Russian Federation had the highest educational level with only 0.5% with no formal education and over 20% with a college degree or higher.

**Table 1:** Sociodemographic characteristics of the study samples, by country (SAGE Wave 1)

	China	Ghana	India	Mexico	Russian Federation	South Africa	Total
Sample size	13,157	4,305	6,560	2,318	3,938	3,836	34,114
Gender							
Male	49.8	52.4	51.0	46.8	41.9	44.1	47.2
Female	50.2	47.6	49.0	53.2	58.1	55.9	52.8
Residence							
Urban	47.3	41.1	28.9	78.8	70.1	64.9	50.4
Rural	52.7	58.9	71.1	21.2	29.9	35.1	49.6
Age Group							
50-59	44.9	39.7	48.6	48.1	44.1	49.9	45.8
60-69	31.9	27.5	30.9	25.6	26.7	30.6	29.7
70-79	18.6	23.1	16.0	17.8	21.4	14.0	18.7
80+	4.6	9.7	4.5	8.6	7.7	5.5	5.8
Education Level							
No formal education	23.1	54.0	51.2	17.2	0.5	25.2	23.8
Less than primary	18.9	10.4	10.0	38.4	1.2	24.0	10.1
Primary school completed	21.0	10.9	14.8	24.0	5.3	22.4	13.5
Secondary school completed	19.9	4.0	10.2	9.9	17.9	14.2	16.0
High school completed	12.6	17.1	8.6	2.4	54.3	8.4	26.2
College completed	4.4	3.4	3.4	5.5	20.7	3.9	9.9
Post graduate degree completed	0.1	0.2	1.7	2.6	0.1	1.8	0.6
Income Quintile							
Lowest	16.3	18.2	18.2	15.3	13.3	20.7	15.9
Second	18.1	19.1	19.5	24.7	17.1	19.9	18.2
Third	20.5	20.5	18.8	16.8	19.6	18.2	19.6
Fourth	23.4	20.7	19.6	16.6	22.1	19.8	21.7
Highest	21.8	21.6	23.9	26.6	27.8	21.3	24.5
Tobacco use							
Never smoker	64.2	75.5	45.5	60.7	65.4	67.7	59.4
Not current smokers	6.6	14.2	4.7	19.1	13.2	9.5	8.5
Current smokers, not daily	2.5	2.6	2.9	6.9	2.0	3.4	2.5
Current smokers, daily	26.7	7.6	46.9	13.3	19.4	19.4	29.6
Alcohol							
Life time abstainer	74.2	57.8	92.5	64.3	33.7	84.5	69.8
Non-heavy drinkers	18.2	39.5	6.9	29.3	55.4	11.5	24.3
Infrequent heavy drinkers	1.2	1.2	0.4	6.2	8.5	3.0	2.9
Frequent heavy drinkers	6.4	1.5	0.2	0.1	2.5	1.0	3.0
Fruit and vegetable intake							
Sufficient	64.3	31.1	9.4	18.6	19.0	31.5	38.1
Insufficient	35.7	68.9	90.6	81.4	81.0	68.5	61.9
Physical activity levels							
High	44.4	61.7	52.3	39.6	61.7	28.2	52.5
Moderate	27.3	12.5	22.8	22.4	15.4	12.3	21.2
Low	28.3	25.8	25.0	38.0	22.9	59.5	26.3

The rate of daily smoking ranged from 7.6% (Ghana) to 46.9% (India), frequent heavy drinker was the highest in China (6.4%) and lowest in Mexico (0.1%), and the highest rate of low physical activity was in South Africa (59.5%). Insufficient fruit and vegetable intake was more common in India, the Russian Federation and Mexico (90.6, 81.0 and 81.4%, respectively) compared with China, South Africa and Ghana (35.7, 68.5 and 68.9%, respectively).

The age standardized prevalence of angina ranged from 9.5 % (South Africa) to 47.5 % (Russian Federation). It was higher in women than in men in all six countries. The rates were higher in rural than in urban locations other than in China. Angina rose with age in each country except Mexico, and a slight drop was seen in the highest age group in Ghana, India, Russian Federation and South Africa. The lowest prevalence of angina was found in individuals with the highest household income in China, Ghana, India and Russian Federation, respectively (see Table 2).

**Table 2:** Prevalence of angina by age, sex, residence and income quintiles among adults aged 50 years and older, by country (SAGE Wave 1)

	China		Ghana		India		Mexico		Russian Federation		South Africa	
	%	95%CI	%	95%CI	%	95%CI	%	95%CI	%	95%CI	%	95%CI
Total	9.9	[8.7,11.2]	13.1	[11.5,14.9]	19.6	[16.5,23.0]	13.9	[8.6,21.6]	47.5	[42.3,52.8]	9.5	[8.5,10.5]
Age group												
50-59	5.6	[4.7,6.6]	11.1	[9.1,13.4]	16.1	[13.7,18.7]	17.9	[8.9,32.8]	37.2	[29.5,45.7]	9.1	[7.7,10.6]
60-69	10.9	[9.4,12.6]	14.2	[11.9,17.0]	21.3	[16.7,26.7]	9.1	[6.6,12.3]	46.2	[39.2,53.3]	9.8	[8.2,11.7]
70-79	17.5	[14.7,20.6]	15.2	[12.5,18.4]	26.3	[20.8,32.6]	8.7	[5.6,13.3]	66.1	[57.5,73.8]	10.1	[7.9,12.8]
80+	23.1	[18.5,28.4]	12.9	[9.6,17.1]	22.3	[16.3,29.7]	16.1	[8.0,29.5]	56.1	[33.5,76.4]	8.0	[5.1,12.3]
Sex												
Men	6.9	[6.0,7.9]	9.8	[8.3,11.6]	16.1	[13.0,19.8]	7.5	[4.9,11.5]	44.6	[36.8,52.6]	7.9	[6.6,9.4]
Women	13.1	[11.5,14.9]	16.8	[14.4,19.4]	23.3	[19.7,27.4]	19.5	[10.4,33.7]	49.6	[42.9,56.4]	10.5	[9.3,12.0]
Residence												
Urban	12.3	[10.0,14.9]	9.7	[7.7,12.1]	19.5	[11.8,30.5]	13.8	[7.7,23.7]	47.4	[41.8,53.0]	9.0	[7.9,10.2]
Rural	8.0	[6.9,9.3]	15.3	[13.1,17.9]	19.6	[17.5,21.9]	14.1	[7.9,23.9]	48.0	[37.0,59.2]	10.3	[8.6,12.2]
Income Quintile												
Q1 (Lowest)	11.3	[9.0,14.0]	15.7	[12.4,19.6]	24.9	[19.6,31.1]	19.8	[11.7,31.7]	43.5	[29.9,58.1]	7.6	[5.8,9.8]
Q2	11.3	[9.4,13.5]	14.5	[11.6,18.1]	17.2	[14.0,20.9]	5.6	[3.2,9.8]	58.2	[48.0,67.7]	8.2	[6.4,10.5]
Q3	11.1	[9.3,13.0]	16.0	[12.9,19.6]	23.4	[16.4,32.2]	29.7	[8.3,66.4]	57.3	[42.6,70.8]	11.1	[9.0,13.7]
Q4	8.7	[7.2,10.6]	12.5	[10.0,15.6]	19.1	[14.4,24.7]	11.1	[6.6,17.9]	47.4	[34.6,60.6]	11.5	[9.4,14.1]
Q5 (Highest)	8.2	[6.2,10.8]	7.3	[5.5,9.6]	14.8	[12.3,17.8]	10.5	[5.2,20.3]	37.4	[29.8,45.6]	8.8	[6.9,11.0]

The prevalence of stroke was 6.1% in Russian Federation, which was higher than the other SAGE countries, while India had the lowest prevalence of 2.0%. In Russian Federation, the prevalence of stroke in men was almost twice that of women. Stroke was higher in urban than in rural locations in all six countries. Stroke prevalence tended to increase with age in all SAGE countries, but a slight drop in 80+ age group in Mexico and Russian Federation. In China, the wealthiest older adults had the lowest stroke prevalence (see Table 3).

**Table 3:** Prevalence of stroke by age, sex, residence and income quintiles among adults aged 50 years and older, by country (SAGE Wave 1)

	China		Ghana		India		Mexico		Russian Federation Federation		South Africa	
	%	95%CI	%	95%CI	%	95%CI	%	95%CI	%	95%CI	%	95%CI
Total	3.0	[2.6,3.5]	2.8	[2.3,3.4]	2.0	[1.6,2.5]	4.3	[2.9,6.4]	6.1	[4.5,8.2]	3.8	[3.2,4.4]
Age group												
50-59	1.5	[1.2,1.9]	1.5	[0.9,2.3]	1.5	[1.0,2.1]	2.2	[0.9,5.5]	4.9	[2.6,9.1]	3.5	[2.7,4.6]
60-69	3.4	[2.7,4.3]	3.2	[2.2,4.6]	2.3	[1.5,3.4]	4.9	[3.1,7.7]	4.1	[2.3,7.2]	3.4	[2.5,4.6]
70-79	5.3	[4.4,6.3]	4.0	[2.9,5.6]	2.5	[1.5,4.2]	8.4	[3.8,17.5]	10.7	[6.1,18.0]	4.6	[3.2,6.5]
80+	7.1	[4.7,10.7]	4.0	[2.1,7.4]	3.5	[1.0,11.0]	6.4	[3.3,11.9]	6.8	[3.6,12.6]	5.4	[3.2,9.1]
Sex												
Men	3.5	[3.0,4.2]	2.7	[2.0,3.6]	2.2	[1.6,3.1]	4.5	[2.8,7.3]	8.5	[5.4,12.9]	3.9	[3.1,5.0]
Women	2.6	[2.1,3.1]	2.9	[2.2,3.8]	1.7	[1.2,2.5]	4.1	[2.2,7.5]	4.3	[2.7,6.8]	3.7	[3.0,4.6]
Residence												
Urban	3.7	[3.0,4.6]	4.3	[3.4,5.5]	2.6	[1.8,3.7]	4.8	[3.0,7.4]	6.7	[5.2,8.6]	4.1	[3.4,4.9]
Rural	2.4	[2.1,2.9]	1.7	[1.2,2.4]	1.8	[1.4,2.3]	2.7	[1.4,5.0]	4.6	[1.7,11.7]	3.1	[2.3,4.3]
Income Quintile												
Q1 (Lowest)	3.2	[2.5,4.1]	1.8	[1.0,3.2]	1.2	[0.5,2.9]	2.7	[1.4,5.4]	5.3	[2.1,12.6]	4.1	[2.9,5.9]
Q2	3.1	[2.3,4.2]	1.9	[1.2,3.0]	2.0	[1.3,3.1]	4.3	[1.5,11.7]	4.8	[2.7,8.3]	2.6	[1.7,4.1]
Q3	3.2	[2.5,4.0]	2.8	[1.8,4.4]	2.6	[1.5,4.5]	6.7	[3.2,13.5]	6.9	[3.4,13.4]	4.3	[3.0,6.1]
Q4	3.5	[2.8,4.4]	2.9	[1.8,4.7]	1.5	[0.9,2.4]	5.0	[2.5,9.8]	5.5	[2.6,11.3]	4.1	[2.9,5.7]
Q5 (Highest)	2.3	[1.6,3.2]	4.2	[2.9,5.9]	2.5	[1.7,3.8]	3.2	[1.3,7.7]	7.1	[3.4,14.4]	3.7	[2.5,5.3]

Table 4 shows the Odds ratios for likelihood of angina by risk factors. Hypertension was associated with angina in China, India and Russian Federation after adjustment for age, sex, urban/rural setting and education (OR ranging from 1.32 [1.13-1.55] in India to 3.80 [2.91-4.96] in Russian Federation). Low and moderate physical activity was also associated with angina in China (ORs were 1.46 [1.22-1.76] and 1.66[1.39-1.99], respectively). Obesity had a stronger association with angina in Russian Federation and China (ORs were 1.48[1.08-2.02] and 1.24[1.01-1.53], respectively). Smoking was associated with angina in India and South Africa (ORs were 1.56[1.02-2.36] and 2.11 [1.23-3.61], respectively). Non-heavy drinking was a protective factor for angina in China (OR was 0.67[0.51-0.87]). The OR (1.56[1.19-2.05]) for insufficient fruits and vegetables intake was highest in Ghana. Household income was associated with reduced odds ratios of angina in China, India and Russian Federation, however higher household income was a risk factor of angina in South Africa (see Table 4).

**Table 4:** Odds ratios* for likelihood of angina by risk factors in six LMICs, SAGE Wave 1

	China			Ghana			India			Mexico			Russian Federation			South Africa		
	OR (95%CI)	*p* value	*p* value for trend	OR (95%CI)	*p* value	*p* value for trend	OR (95%CI)	*p* value	*p* value for trend	OR (95%CI)	*p* value	*p* value for trend	OR (95%CI)	*p* value	*p* value for trend	OR (95%CI)	*p* value	*p* value for trend
Household wealth quintile																		
Q1 (Lowest)	ref.			ref.			ref.			ref.			ref.			ref.		
Q2	0.92 [0.73 - 1.16]	0.472		0.94 [0.66 - 1.33]	0.733		0.62 [0.48 - 0.79]	0.000		0.71 [0.40 - 1.25]	0.232		0.96 [0.66 - 1.39]	0.818		0.84 [0.50 - 1.41]	0.502	
Q3	0.96 [0.77 - 1.20]	0.732		1.05 [0.74 - 1.48]	0.798		0.77 [0.60 - 0.98]	0.034		0.76 [0.41 - 1.39]	0.372		0.90 [0.62 - 1.29]	0.561		1.32 [0.82 - 2.14]	0.255	
Q4	0.66 [0.51 - 0.84]	0.001		0.92 [0.64 - 1.35]	0.683		0.77 [0.60 - 0.98]	0.032		0.77 [0.43 - 1.38]	0.378		0.69 [0.47 - 0.99]	0.047		1.75 [1.07 - 2.87]	0.026	
Q5 (Highest)	0.49 [0.38 - 0.64]	0.000	0.000	0.83 [0.54 - 1.28]	0.402	0.437	0.60 [0.46 - 0.79]	0.000	0.010	0.67 [0.35 - 1.29]	0.228	0.339	0.59 [0.40 - 0.87]	0.007	0.002	1.77 [1.02 - 3.06]	0.042	0.003
Tobacco use																		
Never smoker	ref.			ref.			ref.			ref.			ref.			ref.		
Not current smoker	1.29 [0.90 - 1.84]	0.173		0.39 [0.21 - 0.70]	0.002		1.31 [0.91 - 1.87]	0.141		1.40 [0.77 - 2.55]	0.273		1.39 [0.93 - 2.08]	0.108		2.11 [1.23 - 3.61]	0.007	
Current smoker, not daily	0.82 [0.44 - 1.52]	0.524		0.78 [0.35 - 1.76]	0.558		1.56 [1.02 - 2.36]	0.039		2.03 [0.95 - 4.32]	0.068		0.76 [0.27 - 2.15]	0.610		1.36 [0.64 - 2.87]	0.418	
Current smoker, daily	0.81 [0.63 - 1.05]	0.107	0.092	1.06 [0.70 - 1.59]	0.787	0.757	0.94 [0.79 - 1.11]	0.463	0.530	1.59 [0.82 - 3.09]	0.173	0.069	0.98 [0.67 - 1.43]	0.914	0.555	1.22 [0.81 - 1.85]	0.341	0.422
Alcohol																		
Life time abstainer	ref.			ref.			ref.			ref.			ref.			ref.		
Non-heavy drinkers	0.67 [0.51 - 0.87]	0.003		0.77 [0.59 - 1.01]	0.061		0.90 [0.65 - 1.24]	0.508		0.93 [0.50 - 1.70]	0.803		1.22 [0.92 - 1.61]	0.170		1.37 [0.84 - 2.21]	0.206	
Infrequent heavy drinkers	1.00 [0.45 - 2.25]	0.992		1.26 [0.41 - 3.83]	0.684		2.22 [0.82 - 6.02]	0.118		0.36 [0.08 - 1.62]	0.184		1.18 [0.72 - 1.93]	0.511		1.60 [0.75 - 3.42]	0.221	
Frequent heavy drinkers	0.84 [0.53 - 1.34]	0.473	0.902	0.25 [0.03 - 1.87]	0.176	0.240	1.09 [0.24 - 5.02]	0.915	0.631				1.43 [0.55 - 3.76]	0.463	0.483	0.61 [0.08 - 4.69]	0.632	0.672
Fruit and Vegetable intake																		
Sufficient																		
Insufficient	1.10 [0.89 - 1.34]	0.379		1.56 [1.19 - 2.05]	0.002		0.87 [0.68 - 1.11]	0.267		0.95 [0.58 - 1.56]	0.840		1.28 [0.96 - 1.71]	0.097		0.79 [0.56 - 1.11]	0.179	
Physical activity level																		
High	ref.			ref.			ref.			ref.			ref.			ref.		
Moderate	1.46 [1.22 - 1.76]	0.000		1.42 [1.02 - 1.98]	0.039		1.20 [0.99 - 1.44]	0.060		0.57 [0.33 - 1.01]	0.055		1.08 [0.80 - 1.46]	0.628		1.07 [0.64 - 1.78]	0.808	
Low	1.66 [1.39 - 1.99]	0.000	0.000	0.89 [0.66 - 1.21]	0.460	0.460	1.14 [0.94 - 1.38]	0.182	0.182	0.79 [0.51 - 1.22]	0.285	0.285	1.20 [0.89 - 1.60]	0.228	0.228	0.97 [0.68 - 1.40]	0.889	0.889
Hypertension																		
No																		
Yes	1.79 [1.52 - 2.11]	0.000		1.14 [0.90 - 1.45]	0.281		1.32 [1.13 - 1.55]	0.000		1.01 [0.65 - 1.55]	0.972		3.80 [2.91 - 4.96]	0.000		1.09 [0.75 - 1.60]	0.651	
BMI																		
Normal	ref.			ref.			ref.			ref.			ref.			ref.		
Underweight	0.78 [0.53 - 1.16]	0.216		0.77 [0.54 - 1.08]	0.126		1.14 [0.95 - 1.36]	0.151					1.00 [0.31 - 3.27]	0.998		0.89 [0.38 - 2.07]	0.789	
Overweight	0.94 [0.79 - 1.10]	0.439		1.05 [0.77 - 1.43]	0.770		1.16 [0.93 - 1.44]	0.193		0.83 [0.50 - 1.39]	0.482		1.04 [0.78 - 1.39]	0.803		0.72 [0.47 - 1.11]	0.138	
Obesity	1.24 [1.01 - 1.53]	0.043	0.04	1.05 [0.68 - 1.63]	0.810	0.226	1.28 [0.94 - 1.73]	0.118	0.332	1.39 [0.84 - 2.29]	0.197		1.48 [1.08 - 2.02]	0.015	0.507	1.23 [0.83 - 1.82]	0.294	0.626

*Adjusted for age, sex, urban/rural setting, education and marital status

In all six LIMCs except Mexico, hypertension was associated with stroke (OR ranging from 1.98[1.04-3.80] in Ghana to 3.16[1.72-5.83] in Russian Federation). Low, moderate physical activity were also strongly associated with stroke in four LMICs apart from Ghana and India. In China, Obesity increased the risk of stroke (OR was 1.66[1.20-2.28]). Smoking was also a risk factor of stroke in South Africa. We observed a stronger association between frequent heavy drinking and stroke in India (OR 6.64[1.39 – 31.82]). Insufficient fruit and vegetable intake and household income were not significantly associated with stroke in any of the countries (see Table 5).

**Table 5:** Odds ratios* for likelihood of stroke by risk factors in six LMICs, SAGE Wave 1

	China			Ghana			India			Mexico			Russian Federation			South Africa		
	OR(95%CI)	*p* value	*p* value for trend	OR(95%CI)	*p* value	*p* value for trend	OR(95%CI)	*p* value	*p* value for trend	OR(95%CI)	*p* value	*p* value for trend	OR(95%CI)	*p* value	p value for trend	OR(95%CI)	*p* value	*p* value for trend
Household wealth quintile																		
Q1 (Lowest)	ref.			ref.			ref.			ref.			ref.			ref.		
Q2	1.19 [0.82 - 1.73]	0.352		1.29 [0.47 - 3.57]	0.622		2.04 [0.96 - 4.32]	0.063		1.66 [0.59 - 4.65]	0.338		0.77 [0.39 - 1.52]	0.450		0.44 [0.20 - 0.98]	0.045	
Q3	1.05 [0.71 - 1.54]	0.816		1.48 [0.55 - 4.01]	0.436		1.92 [0.90 - 4.11]	0.093		2.39 [0.86 - 6.62]	0.094		1.44 [0.77 - 2.67]	0.252		0.61 [0.30 - 1.26]	0.181	
Q4	1.25 [0.86 - 1.83]	0.239		1.16 [0.40 - 3.41]	0.786		1.47 [0.67 - 3.21]	0.335		1.87 [0.65 - 5.40]	0.247		0.83 [0.41 - 1.68]	0.602		0.57 [0.26 - 1.23]	0.151	
Q5 (Highest)	0.99 [0.66 - 1.48]	0.953	0.958	1.96 [0.69 - 5.59]	0.210	0.317	2.01 [0.93 - 4.38]	0.078	0.225	1.95 [0.63 - 6.04]	0.246	0.248	0.80 [0.38 - 1.66]	0.548	0.659	0.52 [0.23 - 1.21]	0.129	0.301
Tobacco use																		
Never smoker	ref.			ref.			ref.			ref.			ref.			ref.		
Not current smoker	1.32 [0.81 - 2.13]	0.264		0.76 [0.30 - 1.92]	0.557		1.45 [0.68 - 3.09]	0.333		0.64 [0.25 - 1.63]	0.348		1.55 [0.75 - 3.19]	0.236		1.47 [0.55 - 3.93]	0.446	
Current smoker, not daily	0.67 [0.24 - 1.86]	0.444					0.54 [0.13 - 2.27]	0.401		1.24 [0.40 - 3.86]	0.704					0.96 [0.22 - 4.16]	0.956	
Current smoker, daily	1.06 [0.75 - 1.51]	0.735	0.615	1.37 [0.52 - 3.61]	0.530		1.07 [0.69 - 1.64]	0.77	0.526	0.61 [0.20 - 1.85]	0.384	0.601	1.12 [0.53 - 2.37]	0.769		1.88 [1.02 - 3.48]	0.044	0.237
Alcohol																		
Life time abstainer	ref.			ref.			ref.			ref.			ref.			ref.		
Non-heavy drinkers	0.44 [0.29 - 0.68]	0.000		1.30 [0.70 - 2.41]	0.415		0.41 [0.15 - 1.15]	0.091		1.00 [0.41 - 2.48]	0.992		0.88 [0.51 - 1.51]	0.639		0.76 [0.33 - 1.77]	0.527	
Infrequent heavy drinkers	0.94 [0.33 - 2.65]	0.903					2.46 [0.31 - 19.28]	0.393		2.67 [0.76 - 9.45]	0.127		1.12 [0.46 - 2.74]	0.803		0.96 [0.27 - 3.35]	0.944	
Frequent heavy drinkers	0.47 [0.21 - 1.03]	0.059	0.251				6.64 [1.39 - 31.82]	0.018	0.005									
Fruit and Vegetable intake																		
Sufficient	ref.			ref.			ref.			ref.			ref.			ref.		
Insufficient	1.02 [0.75 - 1.40]	0.882		1.50 [0.77 - 2.92]	0.236		0.84 [0.48 - 1.49]	0.557		0.87 [0.43 - 1.77]	0.711		0.62 [0.38 - 1.01]	0.054		0.74 [0.44 - 1.24]	0.246	
hysical activity level																		
High	ref.			ref.			ref.			ref.			ref.			ref.		
Moderate	1.46 [1.05 - 2.01]	0.023		1.11 [0.46 - 2.67]	0.816		0.58 [0.33 - 1.00]	0.051		1.52 [0.60 - 3.88]	0.381		1.78 [1.02 - 3.11]	0.042		0.99 [0.24 - 4.04]	0.989	
Low	2.33 [1.73 - 3.14]	0.000	0.000	1.90 [0.99 - 3.65]	0.054	0.054	1.34 [0.87 - 2.07]	0.191	0.191	2.33 [1.05 - 5.20]	0.039	0.039	2.20 [1.33 - 3.64]	0.002	0.002	4.79 [2.02 - 11.36]	0.000	0.000
Hypertension																		
No	ref.			ref.			ref.			ref.			ref.			ref.		
Yes	2.08 [1.57 - 2.76]	0.000		1.98 [1.04 - 3.80]	0.039		2.18 [1.49 - 3.19]	0.000		2.07 [0.96 - 4.45]	0.063		3.16 [1.72 - 5.83]	0.000		2.66 [1.20 - 5.93]	0.016	
BMI																		
Normal	ref.			ref.			ref.			ref.			ref.			ref.		
Underweight	0.67 [0.33 - 1.34]	0.252		0.48 [0.16 - 1.41]	0.180		0.98 [0.63 - 1.52]	0.939					2.77 [0.57 - 13.43]	0.207		0.85 [0.24 - 3.06]	0.803	
Overweight	1.07 [0.82 - 1.40]	0.616		1.36 [0.69 - 2.71]	0.373		0.83 [0.49 - 1.41]	0.483		1.39 [0.63 - 3.04]	0.412		0.92 [0.52 - 1.62]	0.770		0.72 [0.35 - 1.48]	0.375	
Obesity	1.66 [1.20 - 2.28]	0.002	0.012	1.19 [0.48 - 2.98]	0.709	0.148	0.45 [0.17 - 1.16]	0.098	0.100	0.99 [0.42 - 2.34]	0.978		1.49 [0.84 - 2.63]	0.175	0.418	1.20 [0.63 - 2.28]	0.577	0.722

*Adjusted for age, sex, urban/rural setting, education and marital status

## Discussion

This study reports the prevalence of two common cardiovascular diseases, angina and stroke, and the relevant risk factors among older adults in six LIMCs. Globally, the age-adjusted CVDs mortality continues to be unevenly distributed: where it has decreased in high income countries(HICs) by 43% in recent decades [19], while LIMCs are drowning in a rising tide of CVD. Although age-standardized rates of death attributable to CVD declined 13% in LMICs from 381 per 100000 in 1990 to 332 per 100000 in 2013, the number of deaths increased 66% from 7.21 million to 12 million in 2013 with ageing and population growth ascribed as the main drivers [19]. Ischaemic heart disease and cerebrovascular disease (stroke) combined accounted for more than 85.1% of all cardiovascular disease deaths in 2016[20]. Our study indicated that CVDs(angina, stroke) were prevalent and variable among older adults in six countries. Angina and stroke were both highest in Russian Federation(47.5%, 6.1% respectively). Women were more likely to have angina than men in all six countries. Stroke was more prevalent in urban than in rural. Angina and stroke both tended to increase with age in China.

Prevalence of CVDs generally appeared to be most closely linked to a country’s stage of epidemiological transition [21], especially when high disease rates in middle age carry through into older ages. Underlying social, environmental, and economic shifts in many countries have led to increasing levels of predominant causes such as tobacco and alcohol use, sedentary lifestyle, unhealthy diets, and suboptimum levels of weight, blood pressure, cholesterol, and plasma glucose. The high and growing prevalence of CVD in LIMCs largely reflects the burden of these key risk factors. Our study revealed that hypertension, high BMI, decreased physical activity, frequent heavy drinking and lower household health were key risk factors of angina and stroke. However, the distribution of risk factors in six counties was unequal, for example, the factor with highest OR of angina in China and Russian Federation was hypertension, whereas it was smoking in India and South Africa.

Hypertension has been shown to be an independent risk factor for acute myocardial infarction and stroke in older people [22, 23]. We found that hypertension was associated with angina in China, India and Russian Federation, in addition it was a risk factor of stroke in five of the six countries in this study (not Mexico). Between 1980 and 2008, blood pressure decreased by 2.0mmHg or more (for men) and 3.5 mmHg or more (for women) per decade in western Europe and Australia but increased by up to 2.7 mmHg over this same period in Oceania, East and West Africa and South and Southeast Asia [24]. Systematic review revealed that blood pressure lowering greatly reduced the major cardiovascular disease events and all-cause mortality, irrespective of starting blood pressure [25].However among these six LIMCs 66% hypertensives were undiagnosed before the survey, 73% untreated and 90% uncontrolled. Although the proportions of undiagnosed and untreated were lowest in Russia (30% and 35%), the uncontrolled rate was higher (83%) [26], low level of health care (primary and secondary prevention) and irregular treatment continued to be a major problem [27]. Hence, further research on early screening strategies, available health care and effective treatment of hypertension may be critical for improving outcomes.

Our study also showed that low physical activity and obesity besides hypertension were both associated with angina and stroke in China, and insufficient fruit and vegetable intake was risk factor of angina in Ghana. Compared with data from 1997, total physical activity in 2009 has decreased by 29% in males and by 38% in females in China [28], and physical inactivity was estimated the third leading risk factor for coronary heart disease [29]. As the relation between physical and obesity well recognized, obesity was an important risk factor of CVD. People are becoming more and more obese. Global age-standardised mean BMI increased from 21.7 kg/m^2^ in 1975 to 24.2 kg/m^2^ in 2014 in men, and from 22.1 kg/m^2^ in 1975 to 24.4 kg/m^2^ in 2014 in women. Over this period, age-standardised prevalence of obesity increased from 3.2% in 1975 to 10.8% in 2014 in men, and from 6·4 to 14.9% in women [30]. More than 50% of the obese individuals in the world lived in just 10 countries (listed in order of number of obese individuals): USA, China, India, Russia, Brazil, Mexico, Egypt, Pakistan, Indonesia, and Germany, and China and India jointly accounting for 15% in 2013[31]. China has moved from 60th place for men and 41st place for women in 1975 to second for both men and women in 2014 in the worldwide ranking of the number of severely obese individuals [30]. Unfortunately, the prevalence of obesity among children and adolescents are both on the rise. In comparison with obesity rate in 1985, it increased by 8.7 times for children and 38.1 times for adolescents [32]. In the World Health Survey 2002-2003, prevalence of low fruits and vegetable consumption among individuals aged 18-99 years in Ghana was the lowest among 52 countries [33]. However, the prevalence was higher (68.9%) among persons aged 50 years and older [34]. We also found that insufficient fruits and vegetables intake was associated with angina in Ghana. All of these contribute to the increasing burden of CVD.

We observed a relationship between smoking and angina, frequent heavy drinking and stroke in India. The prevalence of angina was 19.6% (95%CI:16.5-23.0) in India, the second highest for these six countries. CVD-related conditions contributed nearly two-thirds of the burden of NCD mortality in India [35], with ischemic heart disease(IHD) and stroke contributing substantially to CVD mortality in India (83%) [36].Up to 35% of adults in India consume tobacco [37], with the rate of daily tobacco use was highest(46.9%) among the six LIMCs in this study, highest in younger individuals (20–35 years) [38].The relation between alcohol consumption and CVD has been widely studied. Several analyses showed that low-moderate levels of alcohol consumption had cardio protective effects, while heavy drinking is harmful, usually described as “U-shaped” or “J-shaped” relationship [39, 40]. Aside from alcohol consumption, drinking pattern (binge-pattern drinking) played an important role in elevating the risk of CVD [41, 42]. Another cohort study showed that heterogeneous associations exist between level of alcohol consumption and CVD: compared with moderate drinking, heavy drinking raised risk of coronary death, heart failure, cardiac arrest, ischaemic stroke but a lower risk of myocardial infarction or stable angina [43]. We found that in China non-heavy drinking was a protective factor for angina and stroke, and frequent heavy drinking showed a dangerous effect for stroke in India.

There were a few limitations in our study. Firstly, although SAGE assembled nationally representative cohorts from six countries, the response rates were different across the countries, ranging from 51% in Mexico to 93% in China. The low response rate in Mexico was for specific reasons related to timing of the survey and inability to engage in repeat visits to households to maintain the sample and we note this introduces the potential for selection bias into the results for Mexico. Secondly, the data for stroke and some risk factors were based on self-reports, which may lead to recall bias. However, validated symptom-reporting methods were also used in these analyses to estimate and compare prevalence rates for angina to improve prevalence estimates. Thirdly, the question on stroke in SAGE did not distinguish between ischemic stroke and hemorrhagic stroke. Last, these results are based on cross-sectional data and as such, cannot be sure of the direction of the associations we identified.

## Conclusions

In conclusion, our study provided representative prevalence of angina and stroke and relevant risk factors in elders in six LIMCs. Due to the variation pattern of prevalence and risk factors distribution, policies and health interventions will need to be targeted and tailored for a broad range of local conditions to achieve the health goals set by the United Nations for 2025.

## Abbreviations

BMI: Body mass index
CVD: Cardiovascular disease
GBD: Global burden of disease
GNI: Gross national income
GPAQ: Global Physical Activity Questionnaire
HICs: High income countries
IHD: Ischemic heart disease
LMIC: Low- and middle- income countries
SAGE: Study on global AGEing and adult Health
SES: Social-economic status
WHO: World Health Organization
